# Commercial determinants of health: an approach for strengthening One Health interventions

**DOI:** 10.3389/fpubh.2026.1787079

**Published:** 2026-05-14

**Authors:** Ayako Ebata, Nino Paichadze, Syed Shahid Abbas, Adnan A. Hyder, Gerald Bloom

**Affiliations:** 1 Institute of Development Studies, Brighton, United Kingdom; 2 Center on Commercial Determinants of Health and the Department of Global Health, Milken Institute School of Public Health, The George Washington University, Washington, DC, United States; 3 Boston University School of Public Health, Boston, MA, United States

**Keywords:** AMR, commercial determinants, One Health, pandemic threats, pluralistic health systems, zoonoses

## Abstract

The One Health approach recognizes the interconnectedness of human, animal, and environmental health, yet its implementation has largely focused on biomedical issues such as zoonoses and antimicrobial resistance (AMR). This biomedical focus often overlooks the social and economic drivers that shape these challenges. In this article, we argue that applying a *commercial determinants of health* (CDoH) lens can enhance the analytical and policy relevance of One Health as CDoH defines how non-public actors, activities and incentives support as well as undermine health. Specifically, the CDoH framework enables a systematic analysis of the diverse actors, interests and market dynamics that shape intensified animal production, which creates trade-offs between positive (e.g., economic growth, poverty reduction, food security) and negative (e.g., increased zoonotic risk, AMR, environmental degradation) outcomes. Moreover, it supports engagement with sectors beyond health, helping to identify governance strategies that balance economic, social, and ecological objectives. By integrating CDoH into One Health research and policy, we propose a more context-specific and politically informed approach to managing health risks in a globalized and rapidly changing world.

## Highlights

- One Health strategies have tended to center on direct health outcomes and the regulation of non-health sectors with relatively little recognition of the interests and potential social benefits those sectors provide.
- The formulation of strategies for the governance of One Health can benefit from incorporating the commercial determinants of health approach, which would allow One Health programs to address real world economic, livelihood and political outcomes more explicitly.
- The commercial determinants of health framework provides a tool for analyzing the health impact (positive and negative) of commercial activities and identifying appropriate interventions to improve One Health.

## Introduction

For over two decades, the concept of One Health has aimed to cross boundaries set by academic disciplines, government institutions and economic sectors to improve health for all people, animals and the planet (1). The core idea of One Health is that human health, animal health, and environmental health are intrinsically connected, and that achieving any of the three requires paying attention to the others (2).

Despite the ambition associated with One Health’s multi-sectoral and trans-disciplinary understanding, its operationalization, in practice, has been modest. To date, the One Health concept has been largely applied to issues linked to infectious, particularly zoonotic, diseases (3) and antimicrobial resistance (AMR)—with the aims of understanding the interconnectedness of human, animal and environmental health, and identifying ways to strengthen public policy responses. This dominant application of the One Health concept has been criticized as lacking social, economic and political perspectives that recognize non-biomedical factors that influence people’s behaviors (3).

Building on such criticism of the One Health approach, we argue that considering One Health through the lens of commercial and economic determinants of health (4) will allow researchers to determine how commercial and economic activities impact (positively and negatively) human, animal and environmental health. Below, we outline why this is particularly relevant for issues linked to One Health, and how the commercial and economic lens will help identify what governance arrangements are effective in controlling negative effects of commercial activities (5).

### Commercial determinants of health: the definition and application to One Health

Commercial determinants of health (CDoH, hereafter) define how economically motivated actors, activities and incentives influence health outcomes. We summarize key concepts from Gilmore et al. (4) and Lacy-Nicholas et al. (6) in Table 1.

**Table 1:** Examples of commercial actors, activities and incentives that influence health.

Actors	Activities	Incentives
Private actors ◦For-profit organizations ◦Public companies ◦Private companies ◦Multi- and trans-national corporations Hybrid actors ◦Joint ventures ◦State-owned enterprises ◦Foundations ◦Cooperatives ◦Social enterprises ◦Non-governmental organizations (NGOs) ◦Industry associations Third sector ◦Civil society organizations ◦Voluntary organizations ◦Non-profit organizations ◦Charities	Supply chain activities (e.g., production, sales, input procurement) Marketing and sales practices (e.g., advertisement) Research and development Labor and employment practices Waste management Financial practices Reputational management Political practices (e.g., lobbying)	Financial accountability (e.g., towards investors) Profit maximization Social accountability (e.g., food and nutrition security) Maintaining employment Environmental accountability Cost minimization Regulatory compliance Reputation

Source: Adapted from Gilmore et al. (4) and Lacy-Nicholas et al. (6).

The CDoH concept has so far focused on “harmful” sectors such as tobacco and alcohol, which provide limited health and wellbeing benefits (7). This led to a predominant emphasis on for-profit actors, and action to regulate the use of harmful products. However, as we explore other sectors—e.g. food and agriculture—that provide health risks as well as social and economic benefits, policymakers, practitioners and researchers require multi-sectoral and trans-disciplinary approaches to identify appropriate policy options that balance potential benefits and risks.

### The CDoH framework can help operationalize One Health in three specific ways

First, it provides a framework for analyzing human health issues that have both benefits and harms. Factors that influence One Health can both contribute to and prevent us from achieving social, economic and ecological sustainability. For instance, consumption of animal-source food (ASF) contributes to nutrition security in many low- and middle-income countries (LMICs) where the level of ASF consumption remains low (8). However, excess consumption of ASF is associated with cancer, obesity, and non-communicable diseases (NCDs) (9), and environmental problems (10). In addition, intensive livestock farming increases the risks of emerging infectious diseases, 60% of which originates in animals (11), as well as AMR and environmental pollution (12). But the industrialization of livestock sector—and agriculture more generally—has been associated with positive developmental outcomes—e.g. poverty reduction, improved food and nutrition security, increase employment. The CDoH framework allows researchers to systematically identify positive and negative outcomes, who wins and loses from livestock intensification, and generate policy recommendations that can balance them.

Second, CDoH allows us to unpack the different actors, commercial activities, and interests. As noted by Lacy-Nichols et al. (6), commercial actors are heterogeneous and have different interests (Table 1), which shape how livestock is produced and antimicrobials are used in the farming sector. For instance, our research across Asia has shown that weaknesses in public sector capacity have led to the creation of markets where community members, private companies, and government actors provide veterinary and human health services (13, 14). These actors influence how animals are produced (15) by shaping the livelihoods and business environments. While some activities are largely influenced by profit considerations, we find that many interactions also reflect social mandates in a given community (13). Focusing on understanding commercial dynamics can help identify different interests and actors, and, in turn, entry points for policy change and engagement.

Box 1 **A case study of poultry production systems in Asia**

Asia accounted for 45% of global poultry production in 2024, which has grown at 3.8% annually since 2000 (16). The livestock sector contributes to Asia’s economy: for instance, it accounts for approximately 30% of agricultural GDP in South Asia (16). At the same time, 5,422 zoonotic outbreaks have been identified across Asia’s intensified livestock production systems between 1996 and 2019, and industrial animal farming is associated with a 16–300% increase in risks of zoonotic outbreaks (17).

Employing the CDoH framework, we identify key commercial actors, activities and interests that shape the dynamic poultry production systems in Asia.

**Table:** 

Actors	Activities	Incentives
Private actors ◦Industrial livestock production companies ◦Pharmaceutical companies ◦Finance providers ◦Small-scale livestock producers ◦Farm and processing plant workers Hybrid actors ◦Farmer cooperatives ◦Veterinary associations ◦Industry associations	Input sourcing, farm biosecurity, animal health and environmental management Meat processing and sales Defining wages and working conditions, provision of personal protective equipment Investment and research Compliance to biosecurity, land use, environmental, and public health regulations Lobbying	Profit maximization and cost minimization Social accountability (e.g. community engagement, corporation) Disease and environmental management as enforced by public and social rules

Third, CDoH helps researchers identify the sectors beyond health that influence One Health outcomes. Measures to protect people from increased risks of zoonotic disease and AMR require intervention and governance beyond the health sector. At the global level, this multi-sectoral approach is reflected in the quadripartite representation by the World Health Organization (WHO), World Organisation for Animal Health (WHAO), Food and Agricultural Organization (FAO) and United Nations Environment Programme (UNEP). At the national level, One Health implementation is jointly led by several Ministries such as Health, Agriculture, and the Environment. However, implementation plans tend to focus on human health (18), giving less emphasis to the commercial factors outside health markets that influence decisions. As Box 1 case study demonstrates, dynamics and actors that influence zoonoses outbreaks are predominantly in non-health, commercial sectors. As a result, One Health governance needs to consider the trade-offs and synergies between social, economic and ecological outcomes, including impact on livelihoods and poverty reduction (19, 20), and design policies and governance strategies that can contribute to multiple developmental outcomes, not just health.

## Discussion

The CDoH framework recognizes the complex web of markets that influence people’s health and wellbeing and of the need to identify appropriate policy options. CDoH has been used to understand, for example, how multi-national food companies used marketing and research funding to promote unhealthy foods, increasing NCDs in LMICs (21). Beyond these harmful sectors, CDoH can help identify context-specific and realistic solutions for sectors that produce benefits as well as health harms.

Specifically for One Health, this analytical framework can be used to map actors, activities and incentives influencing animal production, and potential benefits and risks of various policy options to different groups of people. This approach would allow researchers to gain an understanding of commercial interests and dynamics, and in turn could lead to the identification of more realistic strategies for addressing the risks of the emergence of zoonoses and antibiotic-resistant organisms. This is especially important in a context of rapid development and change and growing international concern about the potential danger of future pandemics.

## Data Availability

The original contributions presented in the study are included in the article/supplementary material, further inquiries can be directed to the corresponding author.
